# Alternative treatment of forearm double fractures: new design intramedullary nail

**DOI:** 10.1007/s00402-014-2058-9

**Published:** 2014-07-29

**Authors:** Ahmet Köse, Ali Aydın, Naci Ezirmik, Cahit Emre Can, Murat Topal, Tugay Tipi

**Affiliations:** Department of Orthopaedics and Traumatology, Medical School, Atatürk University, Erzurum, 25240 Türkiye

**Keywords:** Intramedullary nail, Radius, Ulna

## Abstract

**Objective:**

This study aims to evaluate the results of intramedullary nail treatment in surgical treatment of adult displaced radius and ulna diaphyseal fractures.

**Patients and methods:**

Eighteen patients (36 forearm fractures) who underwent intramedullary nail treatment due to radius and ulna fractures were retrospectively analyzed. Adult patients with displaced forearm double fractures were included in this study. Patients with open physeal lines, pathological fractures, Monteggia and Galeazzi fractures, distal radioulnar joint instability, bilateral fractures and bone loss were excluded.

**Results:**

Thirteen patients were male (72.2 %) and five were female (27.8 %). Average age of the patients was 35.16 (18–63). Twelve patients (66.7 %) suffered right and six patients (33.3 %) left forearm fractures. Average follow-up period was 77.7 (55–162) weeks, average bleeding amount was 51.11 (15–100) ml, average time to bone union was 11.3 (8–20) weeks, average surgery time was 61.94 (45–80) min and average fluoroscopy time was approximately 2 (1–5) min. According to Grace-Eversman criteria, results were excellent in 14 (77.8 %) patients, good in 3 (16.8 %) and acceptable in 1 (5.6 %). Average DASH questionnaire score was 15.15 (4–38.8). There was no iatrogenic vascular, neural and bone injury during surgery. There was late rupture of extensor pollicis longus tendon in one patient, 4 months after surgery.

**Conclusion:**

Intramedullary fixation method has advantages, such as closed application, short surgery period, good cosmetic results and early return to movement. We think intramedullary fixation method may be used as an alternative treatment method to plate osteosynthesis in surgical treatment of radius and ulna diaphyseal fractures.

## Introduction

Forearm diaphyseal fractures must be considered as intraarticular fractures due to their functional and anatomical characteristics. Insufficient treatment of forearm fractures negatively affects not only the forearm but also entire upper extremity function [1]. Therefore, in treatment, early mobilization is aimed with providing axial alignment and rotational stability [2]. There is consensus on applying surgical methods in treatment of forearm diaphyseal fractures [3, 4]. Today, the accepted treatment method is plate osteosynthesis [5]. Plate osteosynthesis has high bone union ratios and provides stable fixation. However, it requires extensive surgical exposure and periosteal stripping during application [6, 7]. In recent years, new intramedullary nail designs have been started to be widely used in surgical treatment of forearm structures [1, 3, 4, 8–10]. Intramedullary nail method has advantages such as closed application, less soft tissue injury, cosmetic advantages and providing rotational stability with its locking feature [3, 4].

The aim of our study was to evaluate the results of new design intramedullary radius and ulna nails in surgical treatment of adult displaced forearm double fractures.

## Materials and methods

Informed consents were taken from all of the patients. Ethics committee decision was taken prior to retrospective examination. Standard forearm anteroposterior and lateral radiographs were taken at first admission to the hospital. Arbeitsgemeinschaft für Osteosynthesefragen/Orthopedic Trauma Association (AO/ASIF) system was used for classification of the fractures. Adult patients who had undergone intramedullary nailing for closed displaced radius and ulna fractures were included in the study. Patients with open, pathological, Monteggia fractures, Galeazzi fractures, distal radioulnar joint instabilities, neurovascular injury at first presentation, bilateral fractures, multi trauma and bone loss were excluded.

In this study, eighteen adult patients (36 forearm fractures) with displaced radius and ulna diaphyseal fractures were evaluated. Thirteen patients (72.2 %) were male and five patients (27.8) were female. Average age of the patients was 35.16 (18–63).

Twelve patients (66.7 %) had right forearm fractures and six patients (33.3 %) had left forearm fractures. Etiologically, fractures occurred due to fall in five patients (27.8 %), sports activities in six patients (33.3 %), traffic accidents in six patients (33.3 %), work injury in one patient (5.6 %). Forearm splinting is an option in the first days after surgery in order to alleviate pain in some patients.

Patients who could tolerate the pain were allowed to perform active movements. According to AO/ASIF classification, eight patients (44.4 %) had Type A, eight patients (44.4 %) had Type B and two patients (11.2 %) had Type C fractures. Average hospitalization stay of the patients was 4 (2–7) days. Patients were operated within average of 18 (6–48) h upon admission.

### Design of the new radius-ulna nails

**Fig. 1:**
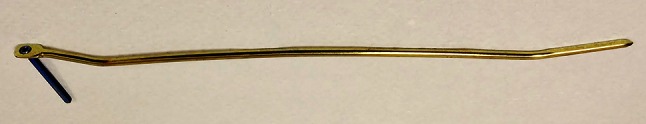
Parabolic shape of the radius nail and view of the locking screw

Radius and ulna nails are made from titanium alloys (TST Rakor Tıbbi Aletler San. ve Tic. Ltd. Sti., İstanbul, Turkey). Radius nail is solid and round. It is a nail which have a parabolic shape which angulates 10° toward anterior in the 3 cm proximal part, which has a distal static locking screw and which provides stability with three-point fixation principal. Distal static locking screw provides a locking with 17° of proximal and volar angle (Fig. 1). This angle prevents the locking screw from directing toward the distal joint surface of the radius. The same radius intramedullary nails can be used for both right and left forearm. Diameter of the nails are 3, 3.5 and 4 mm and length options are 18, 19, 20, 21, 22, 23 and 25 cm. They are used unreamed.

**Fig. 2:**
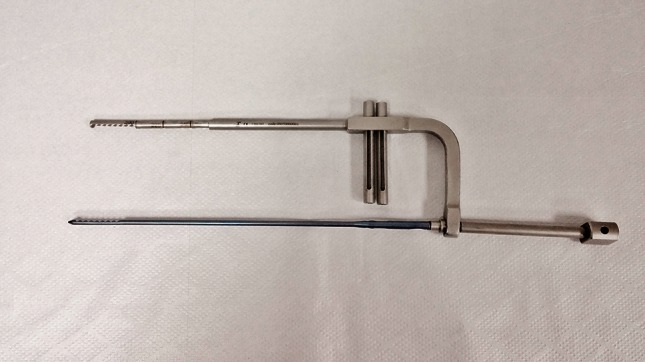
View of the ulna nail over application guide

**Fig. 3:**
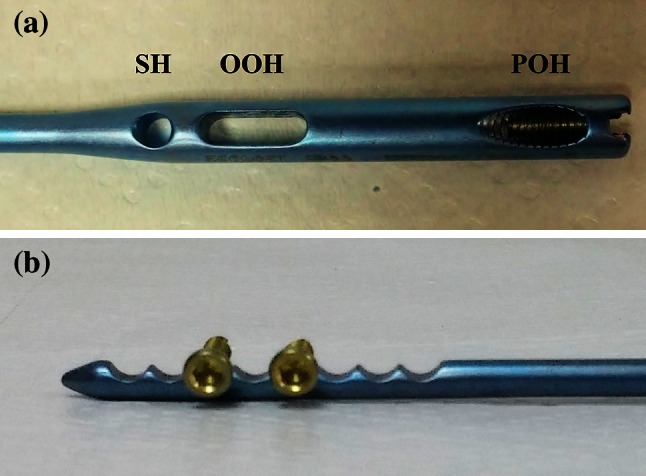
Static locking hole of the proximal of the ulna nail (*SH* static hole), oval oblique hole for compression (*OOH* oval oblique hole), proximal oblique hole for single-cortex locking (*POH* proximal oval hole) (**a**), 8 semi-oval holes on the nail’s distal and view from the locking application (**b**)

**Fig. 4:**
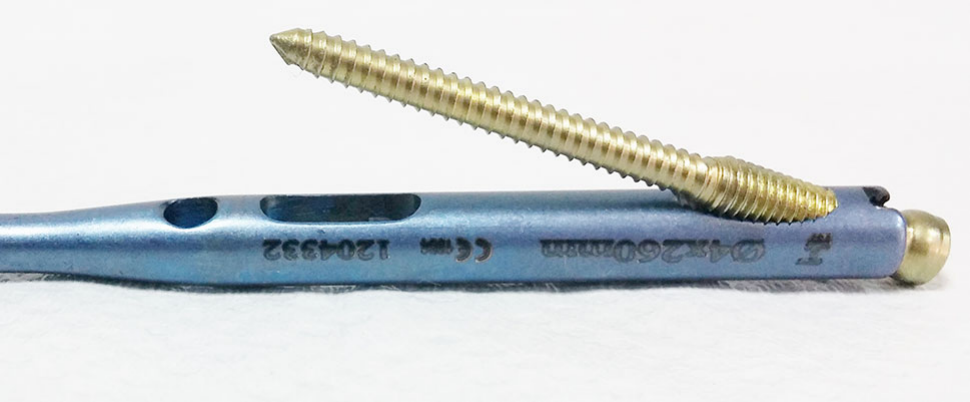
Single-cortex locking through the proximal oblique hole with an angulation of 20°

**Fig. 5:**
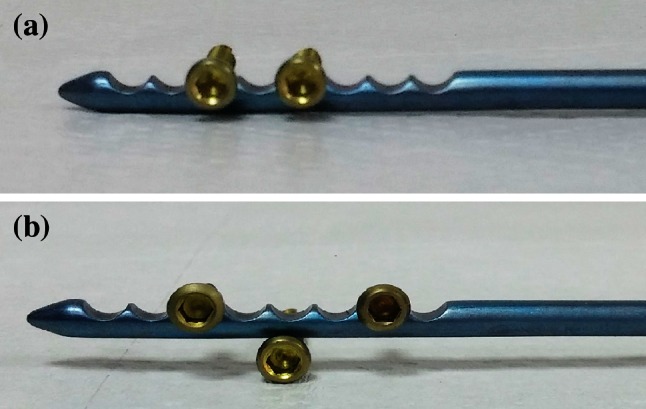
Ulnar distal locking examples (**a**, **b**)

**Fig. 6:**
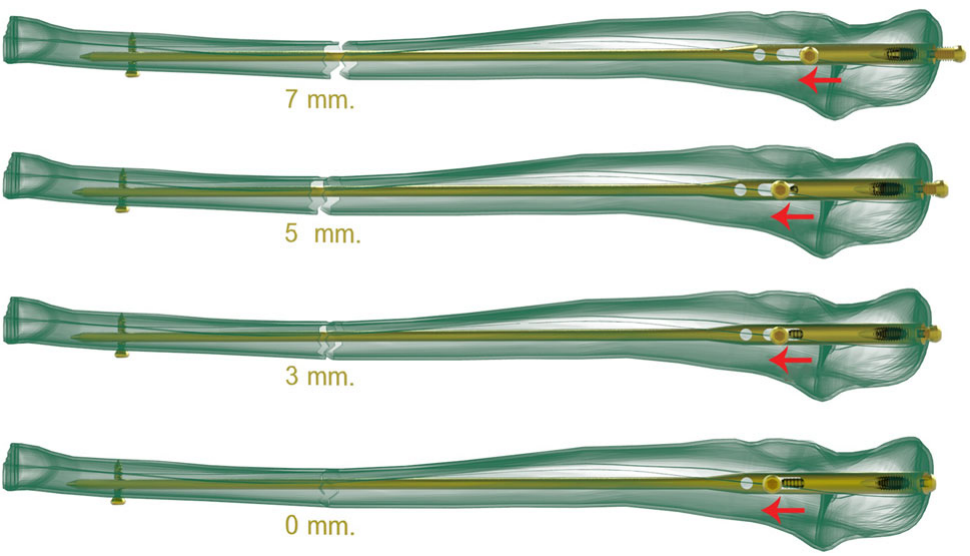
Compression application at the proximal part of the ulna nail

The proximal 4 cm part of the new design locked intramedullary ulna nails is tubular and distal section is in solid form (Fig. 2). Proximal diameter of all nails is 6 mm. In distal section, 3.5, 4, 4.5, 5 and 6 mm diameter choices exist. For nail length, there are 22 different alternatives. Same nail may be used for right and left ulna fractures. Due to its titanium elastic structure, it allows bending with torsional forces. Distal and proximal locking provides rigid axial and rotational fixation. If needed, compression can be done. Intramedullary ulna nail has proximal and distal locking system (Fig. 3). Proximal lock screws may be used in transverse, mediolateral and posteroanterior direction. In proximal locking system; static, single cortex or dynamic locking can be performed through round, oblique or oval holes. Single cortex locking in desired direction can be provided with an angle of 20° from the proximal oblique hole toward the nail axis (Fig. 4). Distal lock allows sufficient number of lockings from 8 semi-oval locking hole in 3 cm distal section of the nail, without requiring a guide and fluoroscopy (Fig. 5). If compression is needed, after providing distal locking with sufficient number of cortical screws, dynamic locking is performed through proximal part of the oval hole. As the compressive top screw is advanced from the proximal part of the nail, it can provide compression over dynamic locking screw in desired amount or up to 7 mm. (Fig. 6) Static locking screw is placed at 4 cm distal to the proximal of the nail. If compression is not required, static locking can be performed through the round hole.

### Surgical technique

Radiographs of the uninjured arm were taken before the operation. Appropriate nails were selected depending on the measurement of the radiographs. The distance between the radial styloid and the radial head’s proximal end were measured. Radial nail length was assessed by 3 cm subtraction from the aforementioned length. Ulnar nail length was assessed by 1.5 cm subtraction from the length between the ulnar styloid and the proximal end of the olecranon. Diameter of the nail depends on the narrowest intercortical distance. To minimize the risk of bias distance between the generator and the detector should be 100 cm. 10 % risk of errors due to inappropriate shooting must be kept in mind while evaluating the radiographs. That is why smaller and larger number of nails should be obtained for the operation. Ulnar nails can be locked statically at the distal end and the proximal end and whereas radial nail can only be locked at the distal end. As the radial nail provides stability according to three points principle the nail with possible bigger diameter which can fill the intercortical space should be selected. Proximal end of the radial nail should be placed in the radial tuberosity. The possible thickest ulnar nail should be used as well. The nail should be placed at the possible most distal position independent of the fracture localization. Distal and proximal locking should be performed afterwards. If there is too much resistance during placement of the nail; to prevent iatrogenic complications during nailing thinner size nail can be used.

Ten patients (55.6 %) underwent regional and eight patients (44.4 %) underwent general anesthesia. Half an hour before surgery, all patients received a single dose of 1 g of cefazolin intravenously. Patients were operated on a radiolucent operation table in the supine position. Fluoroscopy device was placed at the fractured forearm side for reduction control. Closed reduction with use of fluoroscopy was performed in all of the patients. In patients whose stability was ensured with closed reduction, closed operation method was applied. For patients with double forearm fractures, fixation procedure was initiated in the ulna. From the apex of the olecranon, a 2 cm longitudinal skin incision was performed. Insertion of the triceps tendon to the olecranon was passed with longitudinal blunt dissection. A 2-mm-thick K-wire intramedullary was sent from 6.5 mm proximal and 3 mm lateral of the apex of the olecranon [11]. Over K-wire, proximal 5 cm intramedullary section was drilled with a cannulated drill and then the nail was advanced with rotational movements until fracture line. In patients which fixation was performed with closed reduction, nail was sent to the distal end. In patients whom closed reduction was not successful, fixation was provided with limited open reduction. Limited open reduction was done through a 2 cm incision over the fracture site. Limited open reduction provided less soft tissue and periosteal stripping. Distal and proximal locking was performed with the forearm in neutral position. We advise distal locking with the use of distal guide with one or two 3 mm screws. According to status of the fracture, static, single-cortex locking or compression application was performed from the proximal.

Subsequently, radius was operated. With minimum 1 cm proximal of the distal joint of the radius, a 1–1.5 cm longitudinal skin incision from the dorsolateral part of the distal metaphysis (lateral of the Lister’s tubercle) was performed. Lister’s tubercle should be clearly visualized in order to prevent possible tendinous injuries. Extensor carpi radialis longus and brevis tendons were found. Extensor carpi radialis brevis tendon sheath was longitudinally exposed with blunt dissection. Meticulous dissection must be done in order not to injure the tendons. First entry was done with the use of the awl vertical to the radial metaphysis in the second extensor compartment. Depending on the surgeon’s experience and preference, first, second and fourth extensor compartments can also be used as the first entry point. First entry point was widened with bent awl targeting medullary cavity. Radius nail chosen prior to surgery was advanced with the radius holder by using rotational movements. Closed reduction was done when the nail tip reached the fracture line. Following closed reduction, nail intramedullary position was checked with fluoroscopy. Distal end of the nail was advanced until it came in full contact with the metaphyseal cortex and static distal locking was performed.

Rotational alignment must be evaluated during the operation. Physical examination and fluoroscopic evaluation must be done. While advancing the nail through the fracture line reduction should be preserved and checked using the fluoroscopy. Continuity of the outer cortical line should be provided. Range of supination and pronation and flexion and extension at the elbow should be evaluated during the operation. Optimum forearm rotational alignment can be achieved with fluoroscopic guidance and careful examination during the operation.

### Evaluation of the results

Bone union was evaluated according to the lateral and AP radiographs taken during the follow-up. Bridging callus formation was evaluated as union. Hand grip strength of all patients with union was evaluated with hydraulic hand dynamometer (SAEHAN Hydraulic Hand Dynamometer (SH5001), Gyeongnam, South Korea). Separate measurements were taken for treated and healthy forearms, when patients were in sitting position with the shoulders in neutral and abduction, the forearm and wrist in neutral and the elbow in 90° of flexion. In order to prevent muscle fatigue, measurements were done within 3 min intervals and average of three different values was accepted as grip strength. Patients’ wrist, forearm and elbow joint range of motions were measured with goniometer. Functional evaluation was performed according to Grace- Eversman [12] evaluation criteria (Table 1) and DASH (Disabilities of the Arm, Shoulder, and Hand) [13] questionnaire score.

**Table 1:** Grace and Eversmann functional evaluation criteria

	Union	Pronation supination comparison ratio with the uninjured arm
Excellent	+	90–100 %
Good	+	80–89 %
Acceptable	+	60–79 %
Unacceptable	−	<60 %

### Statistical method

Data were analyzed by using SPSS software package. Data were recorded as percentage, arithmetic mean and standard deviation. Compliance of the variables included in the analysis with normal distribution was analyzed with the Kolmogorov–Smirnov test. Spearman’s correlation analysis was used for correlation between parameters. Correlation between pronation, supination and grip strength of the treated and healthy forearms was evaluated with Mann–Whitney *U* test. Correlation between the grip strength, pronation, supination and DASH of the treated forearm was evaluated with Spearman’s correlation analysis. *p* < 0.05 value was considered as the significance level in evaluation of the results.

## Results

**Fig. 7:**
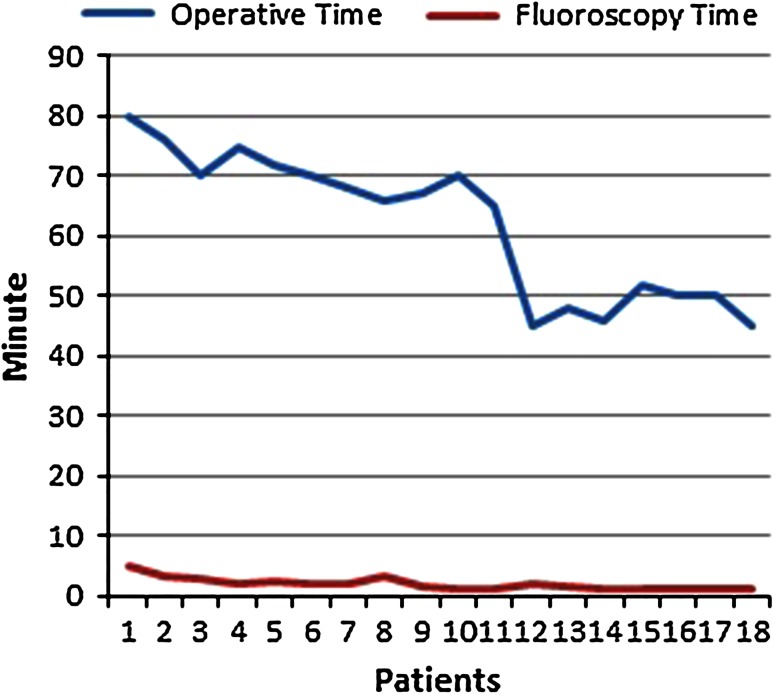
Surgery time with the learning curve and fluoroscopy time distribution based on patients

Average follow-up period was 77.7 (55–162) weeks. Average bleeding amount during surgery was 51.11 (15–100) ml. Average time to bone union was 11.3 (8–20) weeks. Average surgery time was 61.94 (45–80) min and average fluoroscopy time was approximately 2 (1–5) min (Table 2). Changes in the surgery and fluoroscopy times were followed up with learning curve (Fig. 7).

**Table 2:** Comparison of data from studies on forearm nail applications and our study

	Lee et al. [5]	Özkaya et al. [31]	Hong et al. [4]	Bansal [23]	Our study
Follow-up (week), average (range)	Not reported	Not reported	13 months	28 months	77.7 (55–162)
Fluoroscopy time (min), average (range)	7	Not report	Not report	3.5 (2–10)	2 (1–5)
Surgery time (min), average (range)	43 (35–70)	61 (35–90)	78 (28–107)	35 (20–50)	61.94 (45–80)
Time to bone union (week), average (range)	14 (9–32)	10 (9–12)	10 (7–12)	16 (12–28)	11.3 (8–20)
Post follow-up ROM (°)					
Supination	81 (70–88)	Not report	62 (0–96)	Nearly full	73.72 (65–77)
Pronation	79 (68–84)	Not report	80 (0–105)	Nearly full	83.72 (74–90)
DASH score, average (range)	15 (5–61)	13 (3–25)	19 (4–72)	14 (8–36)	15.15 (4–38.8)
Grace-Eversman score					
Perfect	22 (81 %)	18 (90 %)	10 (55 %)	11 (91.7)	14 (77.8 %)
Good	3 (11 %)	2 (10 %)	3 (17 %)	1 (8.3)	3 (16.8 %)
Fair	2 (8 %)		3 (17 %)		1 (5.6 %)
Poor			2 (11 %)		
Grip strength (kgw), average, (SD)					
Treated forearm	Not reported	Not reported	Not reported	Not reported	53.16 (30–90)
Healthy forearm	Not reported	Not reported	Not reported	Not reported	58.6 (35–97)
Bleeding during surgery (ml), average (range)	Not reported	0	60 (20–240)	Not reported	51.11 (15–100)
Complication	3.7 %	10 %	22 %	8.3 %	5.6 %

According to Grace-Eversman criteria evaluation which was performed on bone union and functional results of the patients, results were perfect in 14 (77.8 %) patients, good in 3 (16.8 %) patients and acceptable in 1 (5.6 %). Mean DASH questionnaire score was 15.15 (4–38.8).

In seventeen (94.4 %) patients closed reduction was successful and in one (5.6 %) patient reduction is done with limited open reduction. There was no iatrogenic vascular, neural or bone injury during surgery. Late rupture of the extensor pollicis longus tendon occurred in one patient 4 months after surgery due to an application and technical error.

Patients were applied splint immobilization for an average of 3.6 (2–5) days as they could tolerate the pain. Patients who could tolerate the pain were allowed to perform active movements. There was no patient without bone union and none of the patients developed malunion.

**Fig. 8:**
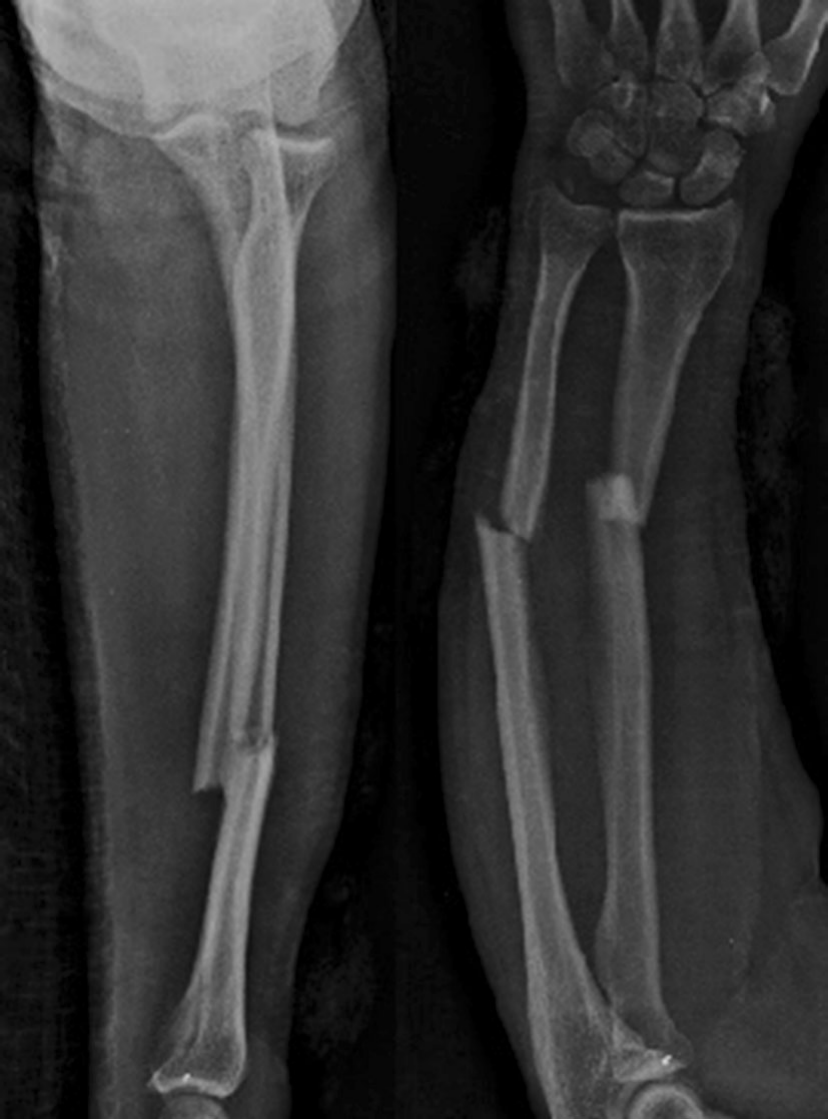
Thirty-two-year-old female patient, preoperative anteroposterior (AP) and lateral direct radiograph of the AO/ASIF Type 22A3 displaced fracture following a fall

**Fig. 9:**
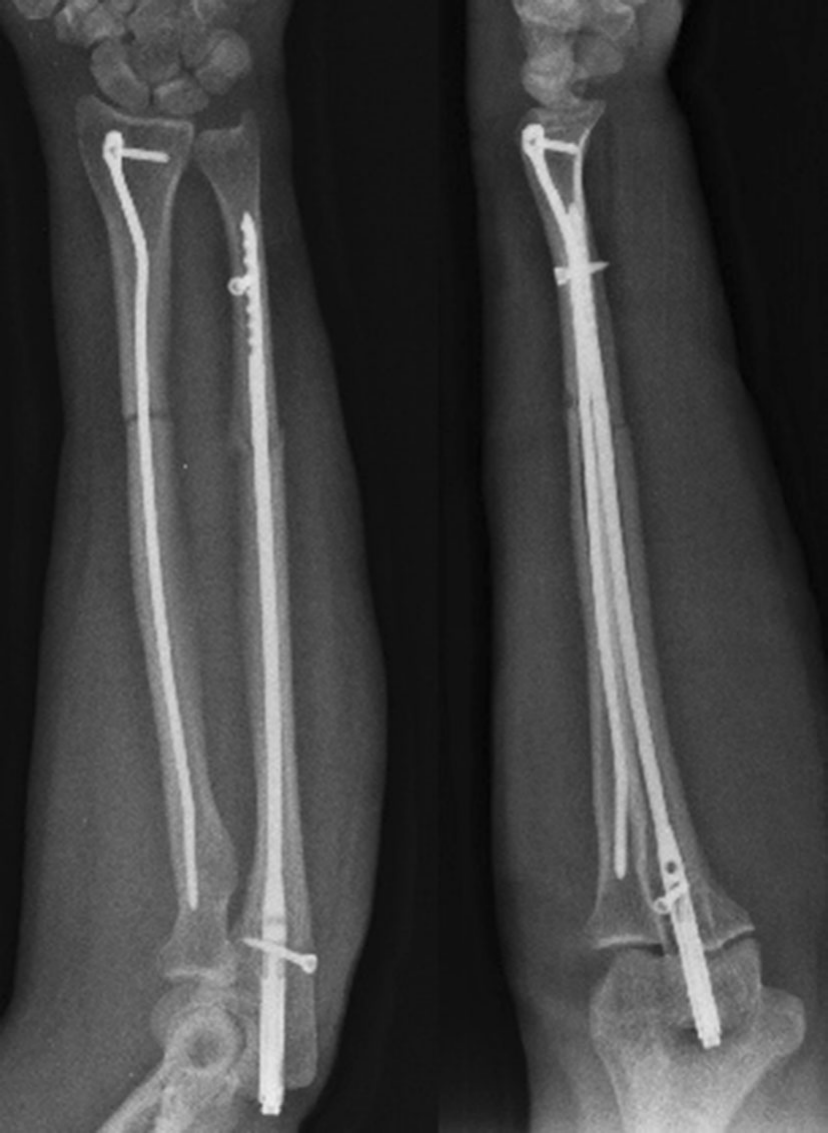
Radial inclination is maintained and compression applied to the ulna fracture line from the proximal can be seen in patient’s postoperative AP and lateral radiograph

**Fig. 10:**
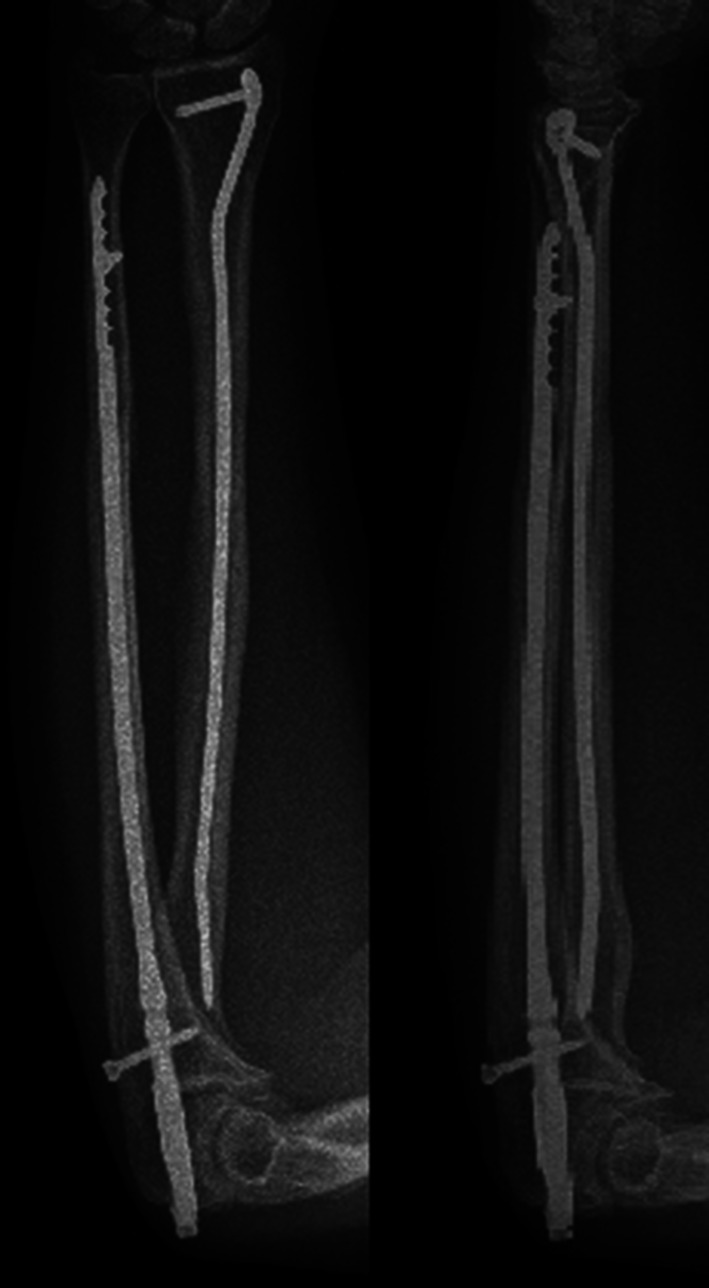
AP and lateral radiograph showing complete union of the radius and ulna fracture after 3 months of surgery

**Fig. 11:**
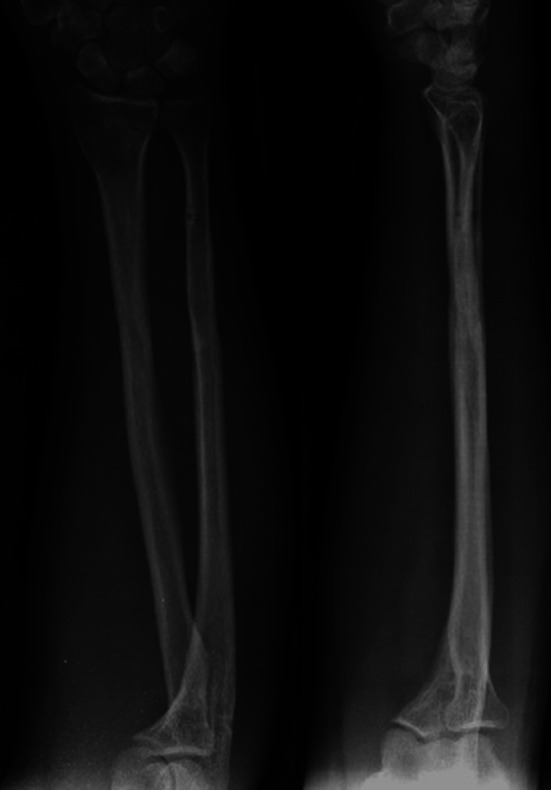
AP and lateral radiographs showing implant removal 20 months after surgery

During the follow-up period, no patient required additional fixation material due to fixation insufficiency. Implant sufficiency, broken implants or mechanic implant irritation findings were not observed. After bone union, implant removal was performed in an average of 18 (4–20) months in three (16.8 %) patients (Figs. 8, 9, 10, 11).

Average elbow flexion of the treated forearm was 142.05° (123°–145°), average elbow extension was 0.66° (0°–5°), average wrist flexion was 73.66° (65°–75°) and average wrist extension was 77.83° (74°–80°). There was no significant difference between the treated and healthy forearm’s elbow and wrist flexion and extension range of motion (*p* > 0.05).

Mean grip strength was 53.16 (30–90) kgw for the treated forearm and 58.66 (35–97) kgw for the healthy forearm. Mean supination was 73.72° (65°–77°) and pronation 83.71° (70°–90°) for the treated forearm (Table 3). Although no difference was observed between the DASH questionnaire score and grip strength of the treated forearm (*p* = 0.302), a negative correlation was found between supination and pronation degrees (Table 4). There was significant difference between the grip strength of healthy and treated forearms (*p* < 0.05). No difference was observed between supination and pronation degrees of the healthy and treated limb (*p* > 0.05) (Table 5).

**Table 3:** Distribution of radiologic and functional values of patients in the treated and healthy forearms

	Treated forearm				Healthy forearm			
	Min	Max	Mean	SD	Min	Max	Mean	SD
Grip strength	30	90	53.16	15.97	35	97	58.6	16.46
Supination	65	77	73.72	3.3	78	80	79.89	0.47
Pronation	74	90	83.72	4.19	90	90	90	0.0

**Table 4:** Correlation among DASH and grip strength, supination and pronation values (Spearman’s correlation analysis**)**

	DASH	
	*r*	*p* ^π^
Grip strength of the treated forearm	−0.238	0.341
Supination of the treated forearm	−0.615	0.007
Pronation of the treated forearm	−0.598	0.009

*r* correlation coefficient

^π^ *p* significance level

**Table 5:** Correlation between radiological and functional results of the treated and healthy forearms (Mann–Whitney *U* test)

	*p*	Mann–Whitney *U*
Grip strength of the forearm (kg)	0.000	000
Supination of the treated forearm (°)	0.302	129.500
Pronation of the treated forearm (°)	0.214	108.500

## Discussion

The best treatment method for diaphyseal fractures of the radius and ulna is plate osteosynthesis which provides open reduction and stable internal fixation [7, 14]. Although the effectiveness of intramedullary nail application as a treatment method is accepted in the tibia, femur and humerus [15], it is not preferred in forearm fractures due to high nonunion ratios and insufficient stability [4]. K-wire, Steinman pin and Lottes forearm nails were used as the fixation materials in first reports regarding the intramedullary treatment of forearm fractures [10]. High nonunion ratios (21 %) reported at the end of the treatment and additional fixation material requirements limited the use of intramedullary nails in forearm fractures [10]. Forearm nails developed in recent years, with perfect functional results and high union rates, have been started to be used in this field [4, 16]. Union ratios between 87 and 98 % were reported in plate screw procedures [17, 18]. In some studies regarding intramedullary nail procedure, union ratios were reported as 92 % by Lee et al. [4] 100 % by Hong et al. [3], 88.6 % by Visna et al. [19], 100 % by De Pedro et al. [16] We obtained a 100 % union in our study.

Although plate fixation provides a high union ratio and safe stable fixation and therefore it is the first treatment procedure which comes to mind in forearm fractures, partially high infection ratios related with soft tissue dissection and periosteal abrasion were also reported [6]. Additionally, mentioned reasons are the factors which affect fracture healing negatively. As intramedullary is performed as a closed procedure, it minimizes the injury to the soft tissue and periosteum. Intramedullary application also affects fracture healing positively as hematoma of the fracture is not discharged [20].

In some studies with intramedullary nail fixation, average time to union was reported as 10 (9–12) weeks by Özkaya et al. [21], 3.5 (2.6–11.6) months by Weckbach et al. [22], 14 (9–32) weeks by Lee et al. [4], 10 (7–12) weeks by Hong et al. [3], and as 15 (10–21) weeks in patients who underwent open reduction. In our study, average time to union was 11.3 (8–20) weeks.

DASH questionnaire score average was 15 (5–61) in Lee et al. [4] and 13 (3–25) in Özkaya et al.’s [21] studies. According to Grace-Eversman [12] criteria, Lee et al. [4] obtained 81 % perfect, 11 % good and 7 % fair results, Özkaya et al. [21] obtained 90 % perfect and 2 % fair results. We obtained 77.8 % perfect, 16.8 % good and 5.6 % acceptable results. DASH questionnaire score average was 15.15 (4–38.8) in our study.

In intramedullary nail procedures, additional fixation materials to ensure the stability of fixation have been used. Sage et al. [10] used long-arm cast for 3 months, Lee et al. [4] used brace for 6 weeks, Hong et al. [3] used splint immobilization for 2–3 weeks for patients with rigid stabilization, and if the stability was not safe, they used long-arm cast until bridging callus formation was observed. Bansal et al. [23] did not perform immobilization. Our intramedullary nails provided axially and rotationally stable fixation with their locking features and the three-point principal. In our series, regardless of stability, splint immobilization was applied to the patients for an average of 2.5 (1–2) days, as they could tolerate the pain. Patients who could tolerate the pain were allowed to perform active movements. Additionally, Crenshaw et al. [1] reported that static locking was not essential in forearm fractures. They suggested that locking decision must be taken based on the rotational stability after nail application. The risk of iatrogenic bone injury is greater in distal ulna due to lesser diameter. Lack of sufficient soft tissue may cause mechanical irritation of the distal ulna [3]. That’s why we advise distal locking with the use of the guide with one or two screws.Static locking was applied to ulna fractures with insufficient rotational stability during surgery.

In treatment of adult forearm both bone fracture starting with which bone is still being debated on [3, 4, 20–23]. There is no evidence regarding the relationship between the priority of treatment and the rotational stability, forearm length and radial bowing in studies about intramedullary nailing of forearm fractures. This subject should be supported with clinical and biomechanical studies. Which bone to begin with depends on the experience of the surgeon. In our study fragmentation and the comminution of the ulna was less so fixation of the ulnae were easier. To preserve length of the forearm it is better to start with the more simple fracture [24]. We think that ulna is the bone to start fixation with in treatment of forearm both bone fractures in adults. Schemitsch and Richards [2] reported that radial inclination and interosseous distance should be maintained. Additionally, they reported that losses which caused 10° or less radical inclination angulation would not create a rotational restriction [25, 26]. If radial inclination and interosseous distance is not maintained, forearm rotation will remain limited. An intramedullary nail of appropriate length and diameter will ensure the continuity of radial inclination due to its titanium elastic feature.

Some problems might be encountered during the intramedullary application. If intramedullary nail diameter is bigger than normal size, it might cause iatrogenic fracture and if the nail diameter is smaller than normal size, it might cause rotational instability [1]. In radius nails with which proximal locking is performed, posterior interosseous branch of the radial nerve is at risk. The extensor pollicus longus tendon and superficial branch of the radial nerve is under risk at the nail application point, in the wrist level [27, 28]. During surgery, iatrogenic vascular, neural, tendon or bone injury was not observed in any patient treated with intramedullary nail fixation due to forearm double fracture. Late rupture of the extensor pollicis longus tendon caused by abrasion developed in a patient with radius and ulna fractures 4 months after intramedullary fixation. Appropriate planning prior to surgery and a controlled and careful approach during surgery will minimize the complications which might occur due to nail preference and surgery technique. The radius nail we use does not have a proximal locking feature, therefore, there is no risk of iatrogenic posterior interosseous nerve damage formation especially in proximal radius diaphyseal fractures caused by locking. Stability is an important issue in forearm proximal diaphyseal fractures. Open reduction and internal fixation possesses certain risks. Exploration of the proximal radius is hard because of abundance of soft tissue coverage and posterior interosseous nerve. The nails with proximal locking screws possess risk for injury to posterior interosseous nerve [29]. The radial nail that we have used had distal and proximal angulation and in between these angulations curvature of the nail was designed to fit to the radius. Parabolic shape and angulated design of the nail provides stability according to the three points principal. Proximal 3 cm angulation of the nail should be placed to the radial tuberosity. That’s why we recommend that the nail can be used for the fracture distal to the radial tuberosity but cannot be used for fracture of the radial head and neck. As there is no risk of neural injury, we think intramedullary nail can be safely used especially in fractures of the proximal 1/3 radius.

Although intramedullary application with closed procedure has advantages such as fracture healing and cosmetic advantages, it also has a disadvantage due to radiation exposure [3, 4]. Seventeen patients (94.4 %) were treated with closed procedure and 1 (5.6 %) patient was treated with limited open procedure.

Removal of internal fixation materials after bone union is a controversial subject [30, 31]. Refracture ratio increases in cases of open, comminuted fractures caused by high-energy traumas, insufficient compression and reduction in comminuted fractures and in case of another fracture in the same limb [30, 31]. Not removing the fixation material for at least 8 months after surgery decreases refracture ratio [31] and refracture might be observed between 2 and 24 months after implant removal [30]. After bone union, implant removal was performed in 3 (16.8 %) patients after an average of 18 (4–20) months. Apart from the patient who developed extensor pollicis longus rupture, no other implant or screw removal was performed due to irritation. Refracture was not observed during patients’ follow-ups.

Our experience with the use of the these nails suggest that the nails should not be used in

1. Patients with open physeal lines
2. Patients with intramedullary diameter less than 3 mm
3. Patients with active infections
4. Patients with radial head and neck fracture
5. Distal ulnar metaphyseal fracture which don’ allow proper locking.

In terms of reliable statistical information, low number of patients and not providing a long-term follow-up after removal of the implant in order to evaluate the refracture risk is a limitation of the study.

## Conclusion

In conclusion, the preferred treatment method for adult forearm fractures is plate osteosynthesis. However, the new-designed forearm nails have advantages, such as application with closed or limited open reduction, short operative time, limited soft tissue dissection of the fracture area in entry points or partial open reduction applications with minimal incision, good cosmetic outcomes, and allowing for early mobilization. Because it has good clinical and functional results, we think intramedullary nail application can be used as an alternative treatment method in surgical treatment of radial and ulnar diaphyseal fractures.
